# The Arthrobacter Species FB24 Arth_1007 (DnaB) Intein Is a Pseudogene

**DOI:** 10.1371/journal.pone.0026361

**Published:** 2011-10-18

**Authors:** Kazuo Tori, Francine B. Perler

**Affiliations:** New England Biolabs, Inc., Ipswich, Massachusetts, United States of America; Louisiana State University and A & M College, United States of America

## Abstract

An Arthrobacter species FB24 gene (locus tag Arth_1007) was previously annotated as a putative intein-containing DnaB helicase of phage origin (Arsp-FB24 DnaB intein). However, it is not a helicase gene because the sequence similarity is limited to inteins. In fact, the flanking exteins total only 66 amino acids. Therefore, the intein should be referred to as the Arsp-FB24 Arth_1007 intein. The Arsp-FB24 Arth_1007 intein failed to splice in its native precursor and in a model precursor. We previously noted that the Arsp-FB24 Arth_1007 intein is the only putative Class 3 intein that is missing the catalytically essential Cys at position 4 of intein Motif F, which is one of the three defining signature residues of this class. Additionally, a catalytically essential His in position 10 of intein Motif B is also absent; this His is the most conserved residue amongst all inteins. Splicing activity was not rescued when these two catalytically important positions were ‘reverted’ back to their consensus residues. This study restores the unity of the Class 3 intein signature sequence in active inteins by demonstrating that the Arsp-FB24 Arth_1007 intein is an inactive pseudogene.

## Introduction

**Figure 1:**
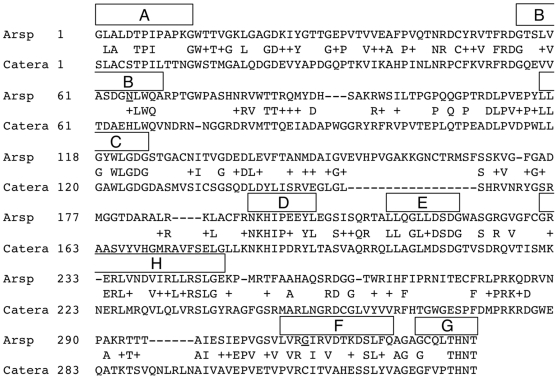
Sequence alignment of the Arsp-FB24 Arth_1007 intein (Arsp) vs. the MP-Catera Gp206 intein (Catera). Conserved splicing motifs (A, B, F, and G) and homing endonuclease motifs (C, D, E and H), as described in the InBase intein database [2], [3], [4], [6], are indicated above the Arsp-FB24 Arth_1007 intein sequence. Positions within each motif are numbered from amino to carboxy terminal and are referred to using the motif letter and the position number separated by a colon. Arsp-FB24 Arth_1007 intein residues Asn^65^ in Motif B position 10 (B∶10) and Gly^311^ in Motif F position 4 (F∶4) are underlined. Residues present in both inteins are listed and similar substitutions are marked with a plus sign.

Inteins are protein splicing elements that remove themselves from host proteins (exteins) during post-translational processing. Intein-mediated protein splicing does not require any exogenous enzymes or cofactors. Inteins are recognized as insertions within other genes and their protein products. They share four conserved splicing domain motifs (A, B, F and G, Figure 1) [1], [2], [3], [4], with many of the most highly conserved residues playing catalytic roles [1], [5], [6], [7]. Some inteins are chimeric proteins with a centrally located homing endonuclease domain containing four endonuclease motifs (C, D, E, and H, Figure 1) [2], [3], [4]. The His at position 10 of intein Motif B (B∶10) is the most conserved intein amino acid (aa) and is essential for splicing in all inteins previously tested [1], [5], [6], [8], [9], [10], [11]; however, it is an Asn in the Arthrobacter species FB24 (Arsp-FB24) Arth_1007 (DnaB) intein. Only this intein and the Thermococcus kodakaraensis KOD1 (Tko) CDC21-1 intein (and its orthologs) lack a His at B∶10 [6], bringing into question the activity of these inteins and if functional, whether they have evolved to use other residues to compensate for the lack of this catalytically essential His^B∶10^.

**Figure 2:**
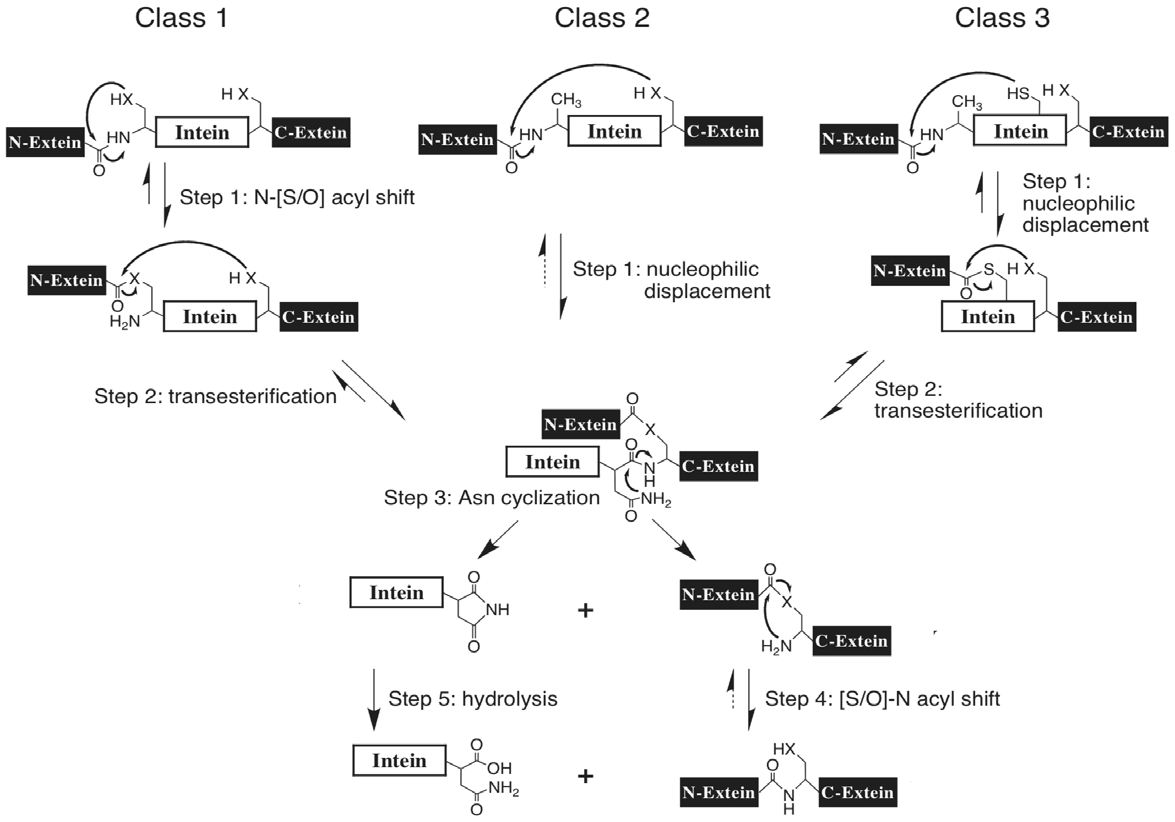
Splicing mechanisms for the three classes of inteins. The Class 1 intein-mediated protein splicing mechanism consists of four coordinated nucleophilic displacement reactions. Class 2 and Class 3 inteins form the same branched intermediate present in Class 1 inteins, but do so by different pathways because they generally start with amino acids incapable of forming the linear (thio)ester intermediate. Once this branched intermediate is formed, the remainder of the splicing reaction is the same in all inteins. Class 3 inteins also form a class specific Motif F branched intermediate with Cys at position F∶4 as the branch point. Residues within the intein assist these enzymatic reactions. However, in all classes there is variability in the residues and positions within the intein that facilitate each reaction. Tetrahedral intermediates are not shown. ‘X’ represents the sulfur or oxygen atom in the side chain of Ser, Thr or Cys. Solid arrows represent steps that have been experimentally verified.

Inteins have been divided into 3 classes based on specific signature sequences and protein splicing mechanisms [1], [10], [11]. Most inteins are Class 1 inteins that splice themselves out of precursor proteins by a four-step mechanism [5], [7], [12] consisting of an initial acyl shift of the intein N-terminal Ser, Thr or Cys to form a linear (thio)ester intermediate, followed by a transesterification reaction that results in a branched intermediate (Figure 2). This branched intermediate is resolved by cyclization of the intein C-terminal Asn, which separates the intein from the ligated exteins. A standard peptide bond is then formed between the exteins by an acyl shift. Class 2 inteins begin with other residues, but their splicing motifs are otherwise similar to Class 1 inteins [6], [13]. Class 1 and Class 2 inteins splice by a mechanism that proceeds through a single branched intermediate (Figure 2) [5], [7], [12], [13].

Class 3 inteins also lack the Class 1 N-terminal nucleophile, but have an additional class specific WCT motif consisting of a Trp at intein Motif B position 12 (B∶12), a Cys at intein Motif F position 4 (F∶4) and a Thr at intein Motif G position 5 (G∶5) [1]. Class 3 inteins splice by a mechanism that includes two branched intermediates (Figure 2), with Cys^F∶4^ being the nucleophile and branch point for the Class 3 specific branched intermediate [1], [10], [11].

The generally accepted assumption is that Class 2 and Class 3 inteins arose from mutation of the N-terminus of a Class 1 intein. To date, all experimental substitutions of a Class 1 intein N-terminus always block splicing if the change is not conservative (Ser, Thr or Cys), so these naturally mutated inteins most likely failed to splice or spliced very poorly unless and until further mutations restored robust splicing. Class 2 inteins solved this problem by overcoming the barrier to direct attack of an amide bond at the N-terminal splice junction by the C-terminal nucleophile (Fig. 2, Step 1 in Class 2 inteins) that is present in all Class 1 inteins tested. Class 3 inteins solved this problem by having Cys^F∶4^ attack the amide bond at the intein N-terminus to form the class specific branched intermediate (Figure 2, Step 1 in Class 3 inteins), which then forms the standard Class 1 intein branched intermediate after a transesterification reaction.

To date, Class 2 inteins have only been found in KlbA genes [6], [13]. Class 3 inteins were previously found to be monophyletic, while other helicase inteins, phage-derived inteins and Class 1 inteins having Cys at F∶4 were polyphyletic [11]. This led to the hypothesis that all Class 3 inteins arose from a phage encoded progenitor intein that lost its N-terminal Ser, Thr or Cys [11]. Based on mutagenesis studies of modern day Class 1 inteins, these early Class 3 inteins would not splice well, if at all. They could have been retained in the population because the extein function was provided by other phage co-infecting the cell or by the host. Eventually, these early Class 3 inteins accumulated second site mutations that enabled them to splice as efficiently as standard Class 1 inteins, as exemplified by the Class 3 Mycobacteriophage Bethlehem (MP-Be) DnaB intein [1], Deinococcus radiodurans (Dra) Snf2 intein [10] and Mycobacteriophage Catera (MP-catera) Gp206 intein [11], which all spliced efficiently in a model precursor consisting of the intein flanked by the Escherichia coli Maltose Binding Protein (M) and the ΔSal fragment of Dirofilaria immitis paramyosin (P).

**Figure 3:**
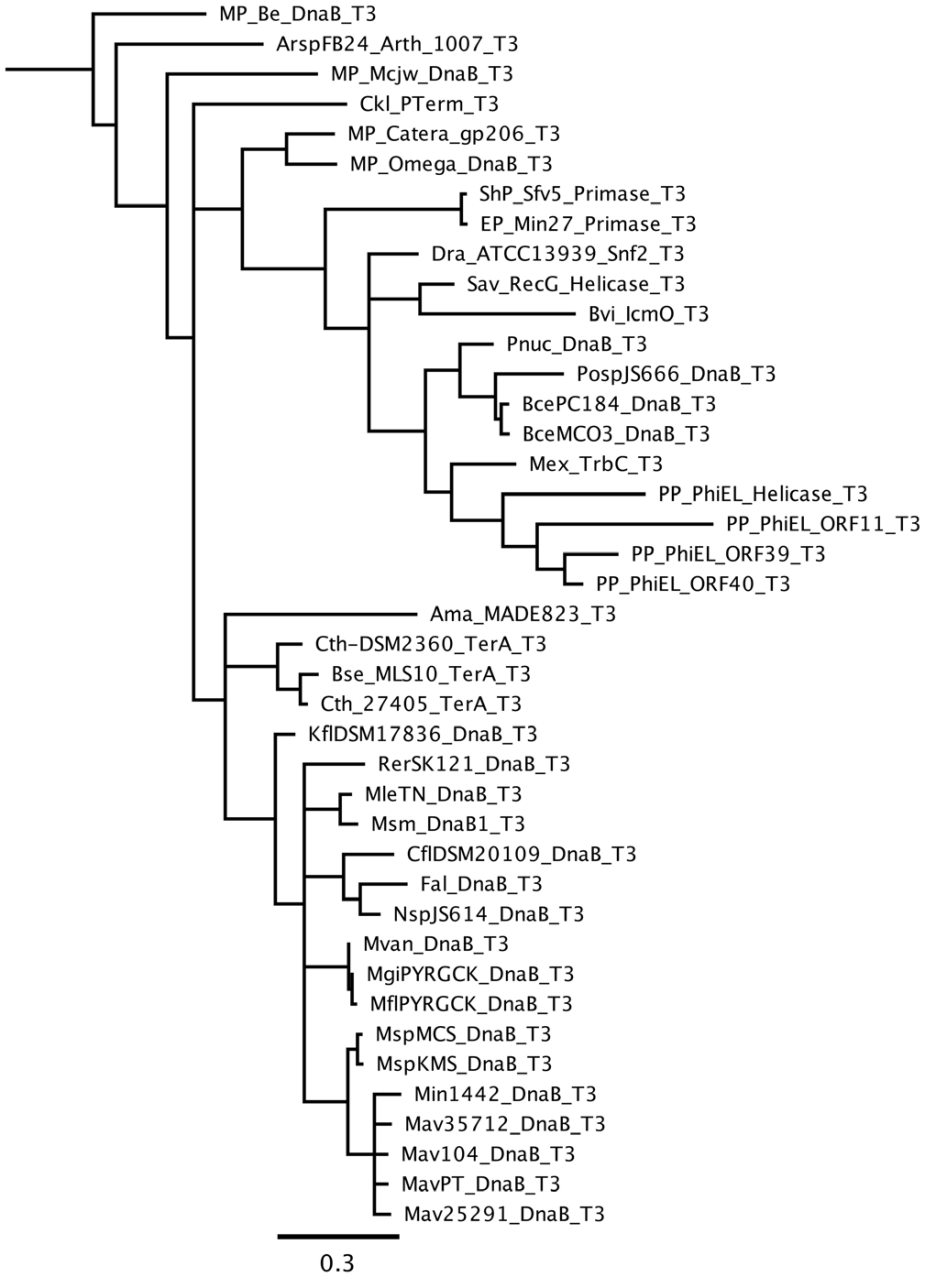
Class 3 inteins are monophyletic. A phylogenetic tree of Class 3 inteins based on conserved intein motifs was generated using MrBayes [15] in the Geneious software package. The scale bar represents 0.3 substitutions per site. A larger phylogenetic analysis previously excluded all non-Class 3 inteins examined from this clade [11]. Except for the Arsp-FB-24 Arth_1007 intein, intein names are defined in the InBase database (http://www.neb.com/neb/inteins.html) [6] with the additional T3 suffix, which indicates that these are Class 3 inteins.

The Arsp-FB24 DnaB intein, which was annotated to be of phage origin [14], is a Class 3 intein based on phylogenetic analysis (Figure 3) and it fulfils all of the sequence criteria listed above for Class 3 inteins except that the catalytically essential Cys^F∶4^ is absent [1], [10], [11]. This suggests that either it is an inactive intein or it is not a Class 3 intein.

## Methods

### Cloning and mutagenesis

All clones were sequenced by the New England Biolabs Core facility and all enzymes were obtained from New England Biolabs (Ipswich, MA) and used as described by the manufacturer. All primers were obtained from Integrated DNA Technologies (San Diego, CA).

The Arthrobacter species FB24 (Arsp-FB24) Arth_1007 intein precursor (locus tag Arth_1007, accession number YP_830503) with an N-terminal His tag was synthesized by DNA2.0, Inc (Menlo Park, CA). Mutations were made in the homing nuclease domain active site to block endonuclease activity (Asp^123^Ala and Asp^219^Ala). The C-extein deletion mutant was constructed using a Phusion™ site-directed mutagenesis kit (New England Biolabs) with appropriate primers that truncated the precursor after the intein C-terminal Asn^332^.

The Arsp-FB24 Arth_1007 intein with 5 native extein residues on both sides was cloned by PCR into a model precursor termed MIP, with the intein flanked by the E. coli Maltose Binding Protein (M) and the ΔSal fragment of D. immitis paramyosin (P) as previously described [1], [10], [11], [13]. All mutations were constructed using the Phusion™ site-directed mutagenesis kit (New England Biolabs) with appropriate primers to introduce the desired mutation.

### Expression, purification, and protein characterization

Precursors were expressed in either the E. coli NEB Turbo strain or NEB Express strain (New England Biolabs) by induction with 0.4 mM isopropyl-β-D-thiogalactoside (IPTG) at OD_600_ of 0.4–0.6 in 10 ml LB medium containing 100 µg/ml ampicillin for 2 hours at 37°C or 15°C overnight. Soluble lysates were boiled for 5 min in SDS Sample Buffer plus DTT (New England Biolabs), loaded onto 10–20% Tris Glycine polyacrylamide gels (Invitrogen, Carlsbad, CA) and either stained with Simply Blue Safe Stain (Invitrogen) or transferred to nitrocellulose membranes for Western Blot analysis with antisera against the Maltose Binding Protein (New England Biolabs), paramyosin or the His tag (Merck, Germany) as described previously [1], [10], [11]. IRDye 680 secondary antibody (Li-Cor Bioscience, Lincoln, NE) was used. The membrane was scanned using an Odyssey infrared imaging system (Li-Cor Bioscience) at 700 nm.

### Phylogenetic analysis

Bayesian inference analysis was performed using the Geneious Pro 5.1 suite of programs (Geneious, Auckland, NZ). Briefly, intein splicing domain Motifs A, B, F and G present in the InBase database (http://www.neb.com/neb/inteins.html) were concatenated to produce a single 49 aa sequence for each intein using names defined in InBase, as previously described [6], [11]. Due to the variable size of Motif F, only the first and last 7 positions of Motif F were included. The concatenated sequences are listed in Tori and Perler [11]. MrBayes [15] was used with default parameters to create trees with final standard deviation of split frequencies of 0.01 or less. The data reported herein represents the Class 3 intein clade, which was found to exclude inteins from all other classes when 148 intein sequences that included all Class 3 inteins, all phage inteins, selected inteins with Cys at F∶4, and selected helicase inteins were previously analyzed [11].

## Results

### Biochemical characterization of the Arsp-FB24 Arth_1007 intein

**Figure 4:**
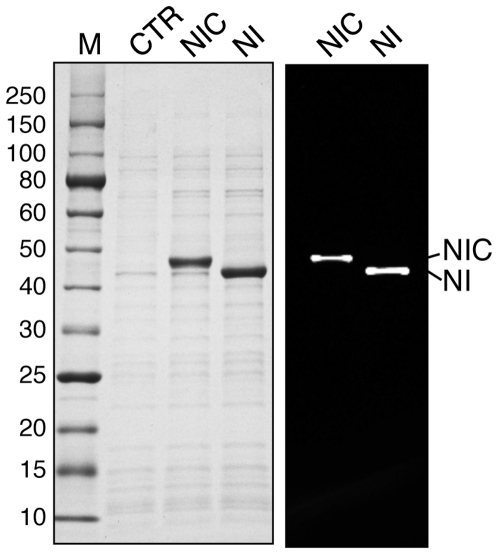
The native Arsp-FB24 Arth_1007 precursor in inactive. The native Arsp-FB24 Arth_1007 precursor (NIC) was expressed in E.coli with the addition of an N-terminal His tag. Because the C-extein is only 14 residues, a precursor truncated at the intein C-terminus (NI) was also expressed. The left panel is a SDS-PAGE of soluble lysates after in vivo expression at 37°C stained with Simply Blue Safe Stain and the right panel is a Western blot of the same samples run in the same gel. Proteins containing the N-terminal His tag were detected with the anti-His tag antibody. The control lane (CTR) contains soluble lysates from E.coli with just an empty plasmid. The sizes of the molecular weight standards (M) are listed in kDa (New England Biolabs 10 to 250 kDa ladder).

The Arsp-FB24 Arth_1007 intein with its complete native N-extein (52 aa) and complete native C-extein (14 aa) was expressed in E. coli at 15 or 37°C. A single band at the predicted size of the precursor (NIC, 44 kDa) was observed in the soluble protein fraction by both staining with Simply Blue Stain or Western Blot with antisera directed against an N-terminal His tag (Figure 4 and data not shown). No spliced product or free intein were observed. As a control, soluble protein samples from a clone expressing an experimentally generated C-terminal cleavage product consisting of the same N-extein and the intein (NI, 42.5 kDa) were co-electrophoresed along with the NIC precursor. NI migrated faster than the complete NIC precursor (Figure 4). No N-terminal (6.4 and 37.7 kDa) or C-terminal (42.5 and 1.5 kDa) single splice junction cleavage products were observed in the NIC precursor sample and no N-terminal cleavage products were observed in the truncated NI precursor sample.

**Figure 5:**
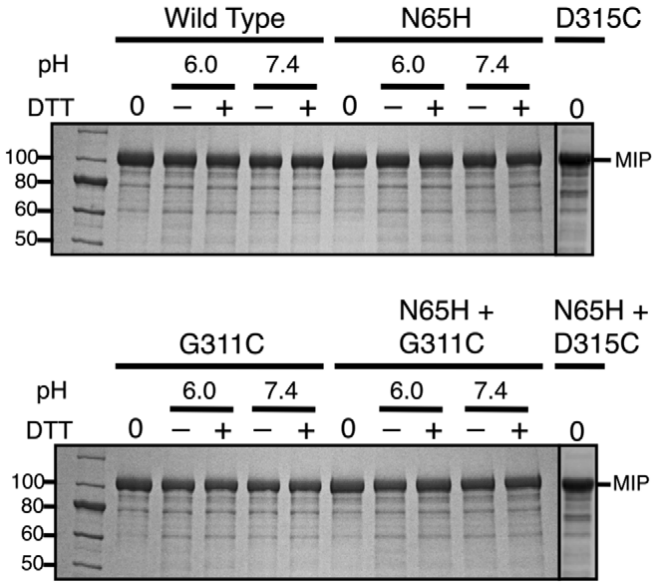
Mutations in the Arsp-FB24 Arth_1007 intein fail to restore activity. Only unspliced MIP precursor (109 kDa) was observed with the wild type intein or with inteins mutated at essential intein residues that restored the consensus amino acid at position B∶10 (Asn^65^) or possible F∶4 positions (Asp^315^ or Gly^311^). Simply Blue Safe stained SDS-PAGE of soluble lysates after in vivo expression at 37°C (0) or after incubation in vitro at 37°C at the indicated pH values in the presence (+) or absence (−) of 50 mM DTT. Western Blots with anti-P sera failed to detect spliced MP or cleavage products in all samples (data not shown). Molecular weight standards are in the left lane of each gel and selected sizes are listed in kDa (New England Biolabs 10 to 250 kDa ladder).

Further characterization was performed on a model precursor (MIP) that has been used to study splicing of numerous inteins because it allows facile identification of splicing and cleavage products that can be clearly differentiated based on relative mobility in SDS-PAGE or immunoreactivity [1], [10], [11], [13]. The Arsp-FB24 Arth_1007 intein with 5 native extein residues on both sides (I) was cloned between the E. coli Maltose Binding Protein (M) and a Paramyosin fragment (P) to generate the Arsp-FB24 Arth_1007 intein MIP precursor. This precursor failed to splice or yield off-pathway cleavage products in vivo when expressed at 15, 30 or 37°C, or when incubated in vitro at 37°C in the presence or absence of 50 mM DTT at either pH 6.0 or 7.4 (Figure 5 and data not shown).

### Mutations that restore missing conserved amino acids

Since His^B∶10^ is essential for splicing in all inteins tested, Asn^65^ (B∶10) was ‘reverted’ back to His. Again, no activity was detected in vivo at 15, 30 or 37°C, or after incubation in vitro at 37°C (Figure 5 and data not shown).

As a putative Class 3 intein, a Cys at position F∶4 in the Arsp-FB24 Arth_1007 intein would be required for splicing [1], [10], [11]. The F∶4 position in the Arsp-FB24 Arth_1007 intein was originally assigned as Asp^315^ [6]. No activity was rescued when Asp^315^ was mutated to Cys alone or in combination with Asn^65^His (Figure 5). The InBase intein-specific BLAST tool (http://www.neb.com/neb/inteins.html) yielded the highest similarity scores between the Arsp-FB24 Arth_1007 intein versus the Mycobacteriophage Omega (MP-Omega) DnaB intein (38% aa identity and 51% aa similarity) and the Mycobacteriophage Catera (MP-Catera) Gp206 intein (35% aa identity and 51% aa similarity) [6], [11]. Based on a pairwise sequence alignment to both of these inteins using both the InBase BLAST tool and BLASTP on the NCBI website (http://blast.ncbi.nlm.nih.gov), the Arsp-FB24 Arth_1007 intein position F∶4 was reassigned as Gly^311^ (Figure 1 and data not shown). However, no activity was observed when Gly^311^ was mutated to Cys or when the Asn^65^His and Gly^311^Cys mutations were combined (Figure 5 and data not shown).

## Discussion

The Arsp-FB24 Arth_1007 intein failed to splice or perform single splice junction cleavage reactions under any condition tested in its native precursor or in a model precursor, even after essential Class 3 intein catalytic residues were mutated back to the consensus residue (His^B∶10^ and Cys^F∶4^). This suggests that the Arsp-FB24 Arth_1007 intein has accumulated other mutations that prevent splicing by directly preventing catalysis or by disrupting proper folding. This is the first case, to our knowledge, of a decayed intein pseudogene that has been retained in a genome. Moreover, the entire precursor is a pseudogene with only a 52 residue N-extein and a 14 residue C-extein. No significant matches to the exteins were found in the NCBI non-redundant database. Therefore, this locus was incorrectly annotated as a DnaB gene based solely on an intein match, pointing out a limitation of automatic annotation and suggesting that intein-containing genes should be checked to insure correct annotation of the extein.

Inteins are usually not detrimental because the intein efficiently removes itself from the host protein during post-translational protein splicing. Surgical removal of the intein is required for extein function because inteins are usually found in highly conserved regions where the presence of the intein disrupts the function of the host protein, such as substrate binding sites and cofactor binding sites [3], [6], [16]. Therefore, the presence of an inactive intein would lead to the accumulation of mutations, deletions and insertions within both the intein and the extein because no selective pressure remains to maintain the correct sequence of the inactive extein or the inactive intein domains. This is exactly what is observed in the Arth_1007 gene encoding the Arsp-FB24 Arth_1007 intein.

No previous examples of decayed inteins have been reported, probably because most inteins are found in essential proteins [3], [6], [16] whose loss would result in reduced viability or cell death. If present in a non-essential extein position, inactivation of such an intein would not be recognizable with time because genetic drift would obliterate intein signatures. The Arsp-FB24 Arth_1007 intein may have been retained in the Arthrobacter species FB24 genome because the prophage genome remnant is irrelevant to survival of the host cell. Alternatively, it may have only recently become inactive and is in the process of being deleted. Another intriguing possibility exists. We previously proposed that Class 3 inteins arose from a single standard phage intein that lost its N-terminal nucleophile [11]. The progenitor Class 3 intein could persist in a phage population until second-site mutations restored splicing efficiency if the extein function was provided by the host cell or by other phage. The Arsp-FB24 Arth_1007 intein could represent a failed intermediate in such an evolutionary progression from the progenitor Class 3 intein.

By demonstrating that the Arsp-FB24 Arth_1007 intein is not functional under any conditions tested, this study resolves the confusion caused by the identification of the Arsp-FB24 Arth_1007 intein as a Class 3 intein based on phylogenetic analysis (Figure 3) and the presence of some Class 3 signature sequence components (Trp at B∶12 and Thr at G∶5) [1], despite the absence of Cys at position F∶4, which performs the mechanistic step that defines Class 3 inteins (Figure 2).

## References

[1] Tori K, Dassa B, Johnson MA, Southworth MW, Brace LE (2010). Splicing of the Mycobacteriophage Bethlehem DnaB intein: identification of a new mechanistic class of inteins that contain an obligate Block F nucleophile.. J Biol Chem.

[2] Perler FB, Olsen GJ, Adam E (1997). Compilation and analysis of intein sequences.. Nucleic Acids Res.

[3] Pietrokovski S (1998). Modular organization of inteins and C-terminal autocatalytic domains.. Protein Sci.

[4] Pietrokovski S (1994). Conserved sequence features of inteins (protein introns) and their use in identifying new inteins and related proteins.. Protein Sci.

[5] Mills KV, Perler FB (2005). The mechanism of intein-mediated protein splicing: variations on a theme.. Protein Pept Lett.

[6] Perler FB (2002). InBase: the Intein Database.. Nucleic Acids Res.

[7] Paulus H (1998). The chemical basis of protein splicing.. Chemical Society Reviews.

[8] Kawasaki M, Nogami S, Satow Y, Ohya Y, Anraku Y (1997). Identification of three core regions essential for protein splicing of the yeast Vma1 protozyme. A random mutagenesis study of the entire Vma1-derived endonuclease sequence.. J Biol Chem.

[9] Romanelli A, Shekhtman A, Cowburn D, Muir TW (2004). Semisynthesis of a segmental isotopically labeled protein splicing precursor: NMR evidence for an unusual peptide bond at the N-extein-intein junction.. Proc Natl Acad Sci U S A.

[10] Brace LE, Southworth MW, Tori K, Cushing ML, Perler F (2010). The Deinococcus radiodurans Snf2 intein caught in the act: detection of the Class 3 intein signature Block F branched intermediate.. Protein Sci.

[11] Tori K, Perler FB (2011). Expanding the definition of class 3 inteins and their proposed phage origin.. J Bacteriol.

[12] Noren CJ, Wang J, Perler FB (2000). Dissecting the Chemistry of Protein Splicing and Its Applications.. Angew Chem Int Ed Engl.

[13] Southworth MW, Benner J, Perler FB (2000). An alternative protein splicing mechanism for inteins lacking an N-terminal nucleophile.. Embo J.

[14] Markowitz VM, Chen IM, Palaniappan K, Chu K, Szeto E (2009). The integrated microbial genomes system: an expanding comparative analysis resource.. Nucleic Acids Res.

[15] Huelsenbeck JP, Ronquist F, Nielsen R, Bollback JP (2001). Bayesian inference of phylogeny and its impact on evolutionary biology.. Science.

[16] Dalgaard JZ, Moser MJ, Hughey R, Mian IS (1997). Statistical modeling, phylogenetic analysis and structure prediction of a protein splicing domain common to inteins and hedgehog proteins.. J Comput Biol.

